# Prevalence of type 2 diabetes mellitus among hepatitis C virus-infected patients: a protocol for systematic review and meta-analysis

**DOI:** 10.1186/s13643-019-0976-x

**Published:** 2019-02-25

**Authors:** Sintayehu Ambachew, Setegn Eshetie, Demeke Geremew, Aklilu Endalamaw, Mulugeta Melku

**Affiliations:** 1 Department of Clinical Chemistry, School of Biomedical and Laboratory Sciences, College of Medicine and Health Sciences, University of Gondar, P.O. Box 196, Gondar, Ethiopia; 2 Department of Medical Microbiology, School of Biomedical and Laboratory Sciences, College of Medicine and Health Sciences, University of Gondar, Gondar, Ethiopia; 3 Department of Immunology and Molecular Biology, School of Biomedical and Laboratory Sciences, College of Medicine and Health Sciences, University of Gondar, Gondar, Ethiopia; 4 Department of Pediatrics and Child Health Nursing, School of Nursing, College of Medicine and Health Sciences, University of Gondar, Gondar, Ethiopia; 5 Department of Hematology and Immunohematology, School of Biomedical and Laboratory Sciences, College of Medicine and Health Sciences, University of Gondar, Gondar, Ethiopia

**Keywords:** Diabetes mellitus, Hepatitis C virus, Protocol

## Abstract

**Introduction:**

The ever-increasing global hepatitis C infection is fueling the burden of diabetes mellitus, which exaggerates various complications and may be a cause of death for millions. Several studies have reported that hepatitis C virus infection is an important risk factor for the development of diabetes mellitus. However, the results of fragmented studies reported variable and inconsistent findings on the prevalence of type 2 diabetes mellitus among hepatitis C virus-infected patients. Therefore, this protocol for meta-analysis will determine the overall pooled prevalence of type 2 diabetes mellitus in patients infected with hepatitis C virus.

**Methods and analysis:**

This systematic review and meta-analysis will include original articles of cohort and cross-sectional studies published in English. A systematic search will be performed in PubMed, Science Direct, Scopus, and Google Scholar. A fixed/random-effects meta-analysis model will be used to estimate the global pooled prevalence of type 2 diabetes mellitus among hepatitis C virus-infected patients. Sensitivity analysis will be conducted to check the stability of the summary estimate. Heterogeneity will be assessed using the *I*^2^ statistic. Subgroup analysis will also be conducted based on geographical region. Funnel plots and Egger’s test and Begg’s test will be used to assess for publication bias.

**Ethics and dissemination:**

The review is based on published data; therefore, ethical approval is not required. The systematic review and meta-analysis will summarize the existing data on the prevalence of type 2 diabetes mellitus among hepatitis C virus-infected patients at the global level. This provides the empirical evidence necessary for researchers, policymakers, and public health stakeholders to derive health-promoting policies, allocate resources, and set priorities for monitoring future trends. The final result will be presented at annual scientific meetings, conferences, and seminars. Moreover, it will also be published in a peer-reviewed reputable journal. We also plan to review every 5 years to provide updated information.

**Systematic review registration:**

PROSPERO CRD42018083409

**Electronic supplementary material:**

The online version of this article (10.1186/s13643-019-0976-x) contains supplementary material, which is available to authorized users.

## Introduction

Diabetes mellitus (DM) and hepatitis C virus (HCV) infection constitute the leading causes of death across the globe [1, 2]. DM causes several complications such as cardiovascular disease, stroke, nephropathy, leg amputation, retinopathy, impaired immunity, and nerve damage, accounting for 8.4% of global all-cause mortality [3]. On the other hand, approximately 150 to 200 million people have been infected with HCV [4, 5]. The global prevalence of viremic HCV is estimated to be 1.0% (95% uncertainty interval 0.8–1.1) in 2015, corresponding to 71.1 million (62.5–79.4) viremic infections [6]. According to some reports, approximately 399,000 people die per year due to HCV-related diseases [7].

Though hepatitis C virus remains a serious public health issue, still no vaccination and no post-exposure prophylaxis are available yet [2]. It causes chronic, life-long infections, resulting in progressive liver damage that leads to cirrhosis and hepatocellular carcinoma [5]. Evidence suggested that slow progression of HCV infections is the major cause of DM; notably, the virus appears to affect glucose metabolism through alteration of the host innate immune response [8]. Some hypotheses suggest that change in carbohydrate and hepatic lipid metabolism, the expression of the HCV core protein, and the activity of hepatic tumor necrosis factor-α induce insulin resistance through the alteration of the insulin receptor substrate signaling pathway [9, 10]. A contemporary review by Desbois et al. has shown that HCV infection is significantly associated with DM/insulin resistance compared with healthy volunteers. Glucose abnormalities are strongly associated with HCV infection and show a negative impact on the main liver-related outcomes [11]. Furthermore, a systematic review and meta-analysis from 33 articles has shown that HCV infection is associated with an increased risk of type 2 diabetes mellitus (T2DM) independently from the severity of the associated liver disease [12]. From 34 eligible studies, the pooled estimators indicated significant DM risk in HCV-infected cases in comparison to non-infected controls in both retrospective (OR adjusted = 1.68, 95% CI 1.15–2.20) and prospective studies (HR adjusted = 1.67, 95% CI 1.28–2.06) [13]. Studies reported that the prevalence rates of T2DM in patients with HCV range from 7.4 to 43.2% [14, 15].

Across the world, several studies were conducted to assess the prevalence rate of T2DM in patients with HCV, having a great disparity and inconsistency in findings. Moreover, there is no previous systematic review and meta-analysis that estimated the global burden of T2DM among HCV-infected patients. This protocol for systematic review and meta-analysis helps to summarize the existing data on the prevalence of T2DM among HCV-infected patients at the global level. This provides pragmatic evidence necessary for researchers, policymakers, and public health stakeholders to derive health-promoting policies, allocate resources, and set priorities for monitoring future trends.

## Objective

This meta-analysis aims to estimate the overall prevalence of T2DM among HCV-infected patients at the global level.

## Review question

This study will answer the following question by summarizing studies published up to December 2017: what is the global prevalence of T2DM among HCV-infected patients?

## Study design and protocol registration

This study entitled “Prevalence of type 2 diabetes mellitus among hepatitis C virus-infected patients: protocol of a systematic review and meta-analysis” is registered online on PROSPERO International Prospective Register of Systematic Reviews (CRD42018083409). Furthermore, the Preferred Reporting Items for Systematic Reviews and Meta-Analyses (Additional file 1) statement guideline [16] will be followed.

## Eligibility criteria for considering studies for the review

### Inclusion criteria

Studies published in peer-reviewed journals, which reported the prevalence of T2DM among HCV-infected patients, will be included. The systematic review will include cross-sectional studies, and any other observational studies which published and contained the minimum information (study participants and number of diabetes cases) that helps to estimate the pooled estimate of the global prevalence will be included. Moreover, studies will be included if DM was diagnosed using (i) fasting plasma glucose (FPG) ≥ 126 mg/dL (7.0 mmol/L) or (ii) 2-h plasma glucose (PG) ≥ 200 mg/dL (11.1 mmol/L) during an oral glucose tolerance test (OGTT), or if a patient with classic symptoms of hyperglycemia or hyperglycemic crisis has (iii) a random PG ≥ 200 mg/dL (11.1 mmol/L) or (iv) a glycated hemoglobin (HbA1c) value of ≥ 6.5%. The full text of studies meeting these criteria will be retrieved and screened to determine eligibility.

### Exclusion criteria

Gestational DM, type 1 DM, non-original research (like review, editorial, and a letter or commentary), and unknown/unclear methods of how DM was diagnosed will not be included. Studies conducted on patients with HCV-HIV or HCV-HBV co-infection and who use antiviral drugs will be excluded. Patients with other causes of liver disease, particularly those known for the development of DM, such as hemochromatosis, will be excluded. Furthermore, patients who have undergone liver or kidney transplantation will not be included in this review.

## Expected outcome

The expected outcome of this study will be the prevalence of T2DM among HCV-infected patients.

## Search strategy

A comprehensive literature search will be conducted to identify studies conducted on the prevalence of T2DM among HCV-infected patients and published up to December 2017 in English. Searches will be carried out systematically in the following electronic search engines: PubMed, Google Scholar, Scopus, and Science Direct. The following keywords will be used to select relevant studies: “Diabetes,” “Diabetes Mellitus,” “type 2 diabetes mellitus,” “type 2 DM,” “T2DM,” “non-insulin dependent diabetes,” “NIDDM,” “hepatitis,” “Hepatitis C,” “hepatitis C virus,” “HCV,” “epidemiology,” and “Prevalence.” An example of the search strategy that will be used in PubMed will be as follows: [(Diabetes Mellitus) OR DM [MeSH Terms]] AND [(Hepatitis) OR (Hepatitis C virus) [MeSH Terms] OR HCV [MeSH Terms]] AND prevalence. The search terms will be used separately and in combination using Boolean operators like “OR” or “AND.” Moreover, a snowball search will also be used to search the citation lists of included studies. Duplicate data will be excluded. The software EndNote version X7 (Thomson Reuters, New York, NY) will be used to manage references and remove duplicated references.

## Quality assessment

Three reviewers (SA, MM, and AE) will independently screen the titles and abstracts to consider the articles in the full-text review. The quality of the studies will be assessed using Joanna Brigg’s Institute quality appraisal criteria (JBI) [17]. The following items will be used to appraise cohort and cross-sectional studies: (1) appropriateness of inclusion criteria, (2) description of study subject and setting, (3) valid and reliable measurement of exposure, (4) objective and standard criteria used, (5) identification of confounder, (6) strategies to handle confounder, (7) outcome measurement, and (8) appropriate statistical analysis.

## Data extraction and management

The authors will extract all necessary data using a standardized data extraction format of Microsoft Office Excel 2016. The data extraction format will include information regarding the country, year of publication, primary author, type of study, study design, study setting, number of participants, age range of the population, diagnostic criteria for each condition, and number of T2DM. Data extraction will be performed by three reviewers (SA, MM, and AE) independently. SE and DG will cross-check for its consistency.

## Data analysis

The STATA version 14 (Stata Corp, 4905 Lakeway Drive, College Station, TX 77845, USA) statistical software will be used for meta-analysis. A fixed/random-effects meta-analysis model will be used to obtain an overall summary estimate of the prevalence across studies. Point estimation with a confidence interval of 95% will be used. Sensitivity analysis will be done to evaluate the effect of each study on the pooled estimated prevalence of T2DM among HCV-infected patients by excluding each study step by step from the analysis process. Publication bias will be assessed by visual inspection of the funnel plots and supplemented with a formal statistical testing using the Egger and Begg tests. Furthermore, the Hoy D et al. [18] tool will be used to assess the risk of study bias. We will apply the trim and fill methods (Duval and Tweedie’s) to test its robustness. The magnitude of heterogeneity across the studies will be evaluated using the *I*^2^ statistic and the Cochran *Q* test. The *I*^2^ provides the percentage of variability due to heterogeneity rather than chance differences and/or sampling error, and the *I*^2^ values of 25%, 50%, and 75% are considered as representing low, medium, and high heterogeneity, respectively. We will perform a subgroup analysis by geographical region, study design, and year that might cause a potential source of substantial heterogeneity.

## Discussion

Hepatitis C virus infection and DM are two major public health problems worldwide. Various studies have found that the prevalence of DM among HCV-infected patients ranges from 7.4 to 43.2% [14, 15]. Studies have shown that several pathways have been involved for the development of T2DM among HCV-infected patients. These include direct viral effects, insulin resistance, pro-inflammatory cytokines, chemokines, and other immune-mediated mechanisms [19]. A systematic review and meta-analysis done by Naing et al. [20] showed an excess risk of T2DM in HCV-infected cases than in non-HCV-infected controls (OR 1.63, 95% CI 1.11–2.39). It is sensible that hepatitis C infection contributes to the rising burden of T2DM. This protocol of the systematic review and meta-analysis aims to estimate the pooled prevalence of T2DM among HCV-infected patients at the global level. The comprehensive estimate result will provide empirical evidence necessary for researchers and decision-makers to draft policy, research needs, and program priorities for the diagnosis, treatment, and management of HCV and DM.

## Strength of the study

This systematic review will provide an inclusive overview of all the fragmented data on the prevalence of T2DM among HCV-infected patients. Being the first systematic review of the published studies reporting on the prevalence of T2DM among HCV-infected patients at the global level, it will provide baseline information for researchers and policymakers. The established clear inclusion and exclusion criteria will provide accurate data for this systematic review. The search will be conducted with no time and geographical area restrictions. This study will adhere to the PRISMA protocols.

## Limitation of the study

Data about the prevalence of T2DM among HCV-infected patients in some countries may not be available. Studies using languages other than English may not be included. Furthermore, the diagnosis of T2DM using different methods may cause heterogeneity across studies.

## Additional file

Additional file 1: This checklist has been adapted for use with systematic review protocol from Table 3 in Moher D et al: Preferred reporting items for systematic review and meta-analysis protocols (PRISMA-P) 2015 statement. (DOC 54 kb)
